# Investigating the Effects of Loading Factors on the In Vitro Pharmaceutical Performance of Mesoporous Materials as Drug Carriers for Ibuprofen

**DOI:** 10.3390/ma10020150

**Published:** 2017-02-09

**Authors:** Junmin Lai, Wu Lin, Peter Scholes, Mingzhong Li

**Affiliations:** 1 School of Pharmacy, De Montfort University, Leicester LE1 9BH, UK; jasminlai0125@gmail.com; 2 Quotient Clinical, Mere Way, Ruddington, Nottingham NG11 6JS, UK; wu.lin@quotientclinical.com (W.L.); peter.scholes@quotientclinical.com (P.S.)

**Keywords:** mesoporous carriers, Syloid^®^ 244FP, Neusilin^®^ US2, poorly water-soluble drugs

## Abstract

The aim of the study was to investigate the effects of the loading factors, i.e., the initial drug loading concentration and the ratio of the drug to carriers, on the in vitro pharmaceutical performance of drug-loaded mesoporous systems. Ibuprofen (IBU) was used as a model drug, and two non-ordered mesoporous materials of commercial silica Syloid^®^ 244FP (S244FP) and Neusilin^®^ US2 (NS2) were selected in the study. The IBU-loaded mesoporous samples were prepared by a solvent immersion method with a rotary evaporation drying technique and characterized by polarized light microscopy (PLM), Fourier transform infrared (FTIR) spectroscopy, X-ray powder diffraction (XRPD) and differential scanning calorimetry (DSC). Dissolution experiments were performed in simulated gastric media at 37 °C under non-sink conditions. The concentration of IBU in solution was determined by HPLC. The study showed that the dissolution rate of IBU can be improved significantly using the mesoporous S224FP carriers due to the conversion of crystalline IBU into the amorphous form. Both of the loading factors affected the IBU dissolution kinetics. Due to the molecular interaction between the IBU and NS2 carriers, the loading factors had little effects on the drug release kinetics with incomplete drug desorption recovery and insignificant dissolution enhancement. Care and extensive evaluation must therefore be taken when mesoporous materials are chosen as carrier delivery systems.

## 1. Introduction

Oral ingestion is the most common route of administration for drugs due to convenience and low cost. However, a major challenge in designing oral dosage forms is poor bioavailability due to many factors, such as drug permeability, aqueous solubility and dissolution rate. Poor water solubility has currently been attributed to almost half of the new molecular entities (NMEs) synthesised annually by pharmaceutical companies [1]. Therefore, improving drug solubility is one of the top priorities in drug product development for the pharmaceutical industry as a whole. Among many different technologies to improve the drug solubility/dissolution rate, such as solid dispersions, nanocrystals, soluble cyclodextrin complexes and self-emulsifying drug delivery systems [1], a delivery approach using mesoporous materials as drug carriers to improve poorly water-soluble active pharmaceutical ingredients (APIs) has shown high potential [2,3]. It has been found that both small and large molecular drugs can be entrapped within the mesopores of the carriers of ordered/non-ordered silica, Al_2_O_3_ or TiO_2_ due to the confined space of the pores of between 2 and 50 nm in diameter, which are only a few times larger than the drug molecules themselves [4,5]. Therefore, the well-ordered crystalline structures of the drug can be suppressed into an amorphous or disordered structure in the mesopores, resulting in the improved wettability and a faster dissolution rate. Furthermore, due to an increased dissolution rate of the amorphous drug, a significantly higher concentration of drug can be generated in the stomach and gastrointestinal tract in comparison with the equilibrium solubility of its stable crystalline form, leading to a higher absorption.

A solvent immersion method is the most common technique for loading drugs into the mesoporous carriers due to its advantages of simplicity and high efficiency [6,7,8,9]. In the immersion method, the porous materials are immersed into a concentrated drug solution for the adsorption of drug molecules on the walls of the pores, and then, the drug loaded mesoporous materials are recovered through different approaches, such as filtration, slow evaporation, spray drying and rotary evaporation [7]. The properties of the mesoporous carriers, including the particle size/morphology, pore diameter, total surface area and volume and surface chemistry, can affect the drug loading efficiency and release rate [3,4,6,9,10,11,12,13,14,15,16]. It has also been found that the loading procedure is a crucial factor affecting the drug loading efficiency, packing of the drug molecules in the pores and thereafter the release kinetics. It was observed that a high drug loading concentration was important to diffuse the drug into the pores in the immersion loading method; however, if most of the drug molecules pack on the surface close to the pore opening, a blockage can happen during the release process due to drug crystallisation on top of the pores, resulting in retardation of the drug release [17]. The ratio of the drug to carrier particles can also affect drug release profiles, with a faster drug release profile obtained at a lower ratio of the drug to carriers [9]. Drying is another crucial stage for the incorporation of drug into the carriers because recrystallization of the drug can occur on the surface of mesoporous particles or as separate crystals during solvent evaporation, leading to a slower drug release rate [18]. Although there is ongoing research into the use of porous materials as drug delivery systems [2,3], there is still a lack of systematic studies to evaluate the drug loading procedure.

In this study, the effects of the loading factors, i.e., the initial drug loading concentration in solution and the ratio of the drug to carriers, on the in vitro pharmaceutical performance of a poorly-soluble drug, ibuprofen (IBU), loaded mesoporous systems were systematically evaluated. In the previous studies, it has been shown that selection of the mesoporous materials significantly affects the drug release performance in the delivery systems [3]. Therefore, in order to identify the effects of the loading factors in different drug delivery systems, two types of mesoporous materials of commercial silica Syloid^®^ 244FP (S224FP) and Neusilin^®^ US2 (NS2) as carriers were compared. S224FP is non-ordered porous silicon dioxide with a neutral pH and has randomly-oriented pores with an average pore size of 19 nm in diameter [19]. The average size of S224FP particles is 5.5 µm with a total surface area of 300 m^2^/g. NS2 is a synthetic, amorphous form of magnesium aluminometasilicate with a neutral pH and an average pore size of 15 nm in diameter [20]. The average size of NS2 particles is 100 µm with a total surface area of 300 m^2^/g. IBU, a weakly-acidic non-steroidal anti-inflammatory drug (NSAID), was chosen as a model compound because of its poor aqueous solubility at a low pH solution [21], which was loaded in the mesoporous carriers in a polar solvent of ethanol. The structures of IBU and mesoporous carriers of S224FP and NS2 are shown in Figure S1 in the Supplementary Materials. In the study, after the solvent immersion process, the drug loaded carrier particles were collected using a rotary evaporator. In comparison with the other drying methods, the utilization of rotary evaporation in the loading process has shown significant advantages, such as process simplification by avoiding filtration to reduce the risk of crystallization of the drug on the surface of the carriers, fixing amounts of the loaded drug and providing industrial scalability for loading the drug into porous materials [7]. Different techniques of polarized light microscopy (PLM), Fourier transform infrared (FTIR) spectroscopy, differential scanning calorimetry (DSC) and X-ray powder diffraction (XRPD) were used to examine and quantify the physical states of the loaded IBU. The IBU dissolution behaviours from the drug-loaded carriers at different loading conditions were compared in pH 2.0 simulated gastric media without pepsin [22] at 37 °C under non-sink conditions in which the level of supersaturation generated by the samples can be evaluated. The desorption recovery of IBU from the carriers was determined in ethanol. The changes of the dissolution behaviours of IBU from the drug loaded samples were also investigated after a three-month storage at room temperature.

## 2. Results

### 2.1. PLM

**Figure 1:**
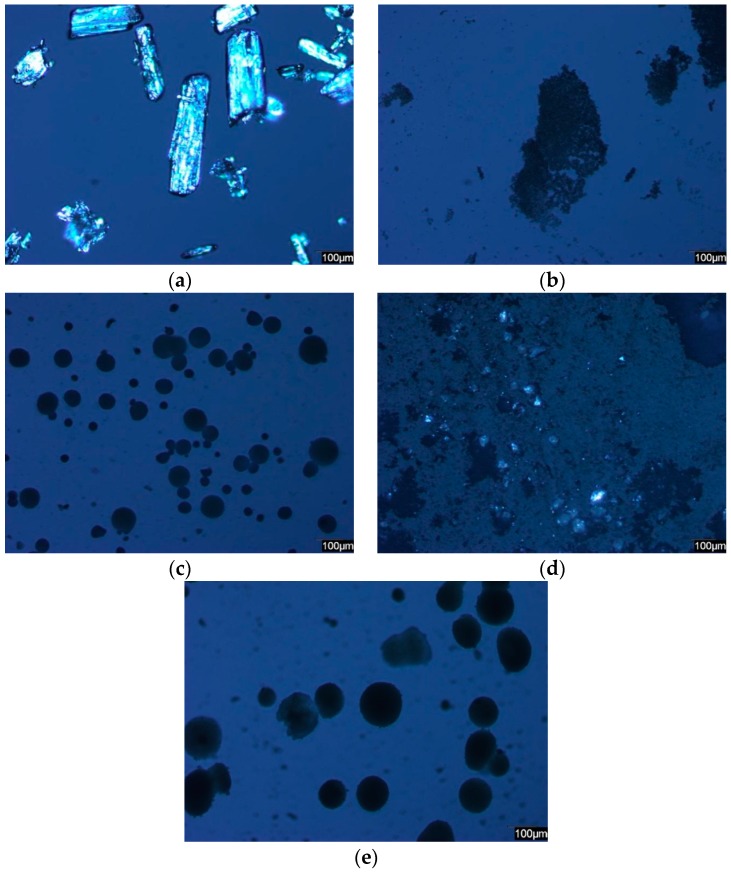
Representative polarized light microscopy (PLM) images of: (**a**) pure ibuprofen (IBU); (**b**) pure Syloid^®^ 244FP (S224FP); (**c**) pure Neusilin^®^ US2 (NS2); (**d**) IBU-loaded S422FP samples; (**e**) IBU-loaded NS2 samples.

Figure 1 shows the PLM images of pure IBU, carriers and representative drug-loaded samples under the same light conditions. The images of the pure crystalline IBU were bright rectangular shapes under the polarized light in Figure 1a. As the average particle size of S244FP was 5.5 µm, the images were tiny irregular dark particles in Figure 1b. NS2 was much larger dark particles with a round shape and wider size distribution in Figure 1c. Bright spots were clearly found in the images of the IBU-loaded S244FP samples, indicating that some of IBU in crystalline form was adsorbed on the outer surface of the carriers in Figure 1d. In contrast, there was no significant difference between the images of the original NS2 particles and the IBU-loaded NS2 samples, indicating that there was no crystalline of IBU on the outer surface of the carriers in Figure 1e. Representative images of each of the drug-loaded samples can be found in Figure S2 in the Supplementary Materials.

### 2.2. FTIR and Drug Desorption Recovery

**Figure 2:**
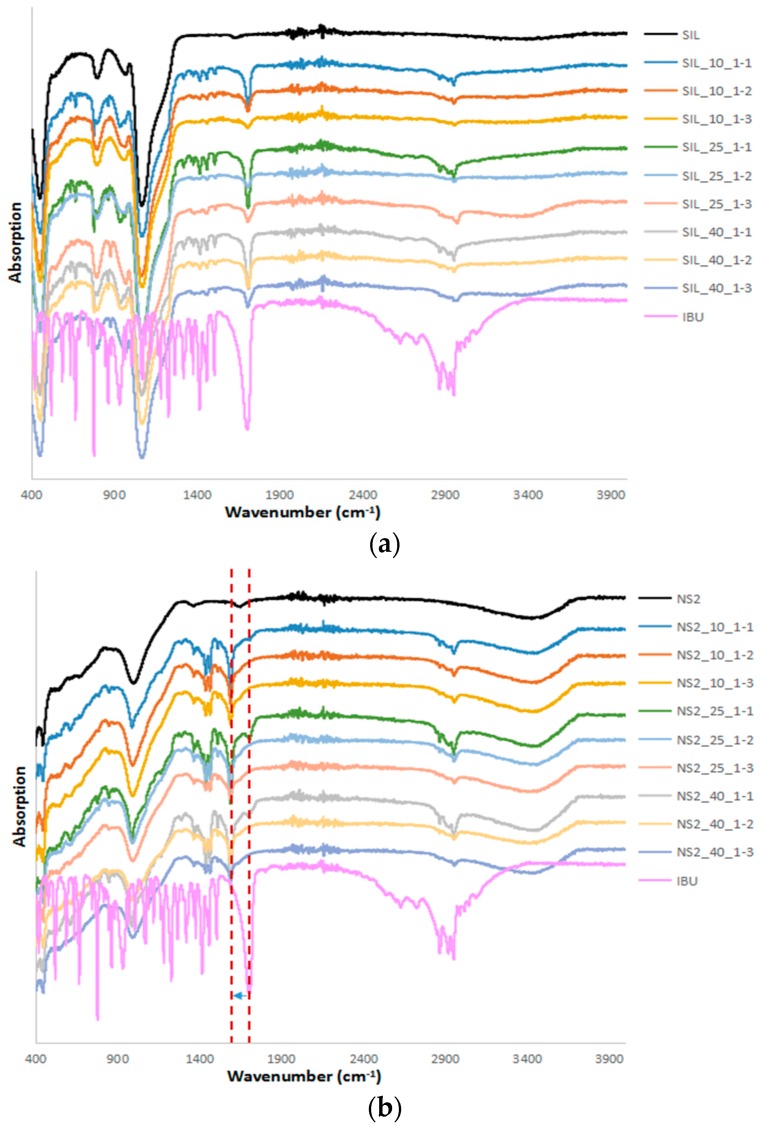
Fourier transform infrared (FTIR) results of drug-loaded (**a**) S244FP and (**b**) NS2 under different drug loading conditions (refer to Table 1 for sample details).

FTIR was used to examine the effect of the loaded drugs on the chemical structure of the carrier particles shown in Figure 2. The FTIR spectra of IBU showed a single characteristic peak at 1711 cm^−1^ due to carbonyl (C=O) stretching and three characteristic peaks between 2800 and 3300 cm^−1^ corresponding to the aromatic benzene ring C–H stretching [23]. S244FP carriers showed a stronger intensity band from 1300 to 900 cm^−1^ due to Si–O stretching of the silanol group [24]. The FTIR spectra of NS2 also showed a broad band from 1300 to 900 cm^−1^ of the Al–O–Si stretching group and a broad stretch band at 3700–3000 cm^−1^ of the O–H stretching of the silanol group [25]. For all of the IBU-loaded S244FP particles, the spectra in Figure 2a showed the combined individual characteristic peaks of IBU and S244FP carriers, indicating that IBU was physically rather than chemically adsorbed on the outer surface of the porous silica particles. In contrast, the carbonyl C=O peak in IBU located at 1711 cm^−1^ was reduced to 1591 cm^−1^ in the spectra of the IBU-loaded NS2 particles, indicating the strong intermolecular interactions between IBU and carriers, which would suggest that the IBU carbonyl group reacted with the OH groups of MgO and Al_2_O_3_ on the surface sites of the porous NS2 particles to form a salt [26]. Because SiO_2_ did not take part in the reaction, the characteristic peak of the silanol group in NS2 at the bands of 1300–900 cm^−1^ and 3700–3000 cm^−1^ did not show any significant shift.

The IBU desorption from the carriers at different loading conditions was determined by extracting the drug-loaded samples using ethanol shown in Table 1, and the desorption recovery was calculated based on the initial ratio of the drug to carrier used in the experiment. A wide range of recovery rates of the IBU-loaded S244FP carriers was obtained from 78% to 109%. No direct correlation between the loading conditions and drug recovery was apparent. Most of the desorption recoveries were between 85% and 100%, indicating that the IBU loading to S244FP carriers is highly reversible with minimal interaction between the drug and the carriers. However, only 40%–54% of IBU the desorption recovery rates were obtained from the IBU-loaded NS2 samples, reflecting the incomplete desorption of drug from the carriers possibly due to the intermolecular interactions, as shown in the FTIR results in Figure 2b. It was also found that there was no correlation between the loading conditions and the IBU desorption recovery from the carriers.

### 2.3. XRPD and DSC

**Figure 3:**
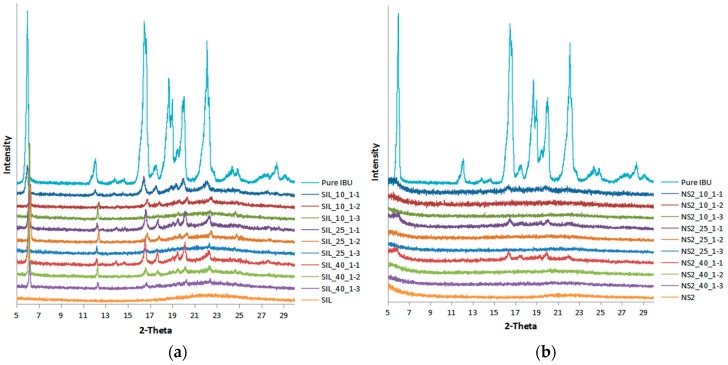
X-ray powder diffraction (XRPD) results of the drug-loaded (**a**) S244FP and (**b**) NS2 under different drug loading conditions (refer to Table 1 for sample details).

The physical state of IBU in the drug-loaded samples was assessed using XRPD and DSC. Figure 3 shows that pure IBU was a crystalline substance with sharp distinct peaks observed at 2θ of 6.0°, 12.1°, 16.5°, 18.7°, 20.1° and 22.1°, which were in good agreement with the previous report [16]. Both S244FP and NS2 carriers were amorphous without any distinctive peak. The characteristic crystalline peaks of IBU were observed in all of the IBU-loaded S244FP samples, indicating that some drug was remaining as the crystalline form in the mesoporous systems under the studied loading conditions. It is possible that some drug adsorbed on the outer surface of the carrier was in crystalline form, an observation further supported by DSC data. However, when the drug to carrier ratio reduced, the characteristic crystalline peaks of IBU decreased, which could be due to the fact that more carrier particles are available for drug adsorption, leading to higher levels of drug being inside the mesopores in the amorphous form. In contrast, there was the complete disappearance of diffraction peaks of IBU detected in the XRPD patterns for all of the IBU-loaded NS2 particles at the drug to NS2 ratios of 1:2 and 1:3, indicating that IBU both inside of the pore and adsorbed on the outer surface of materials can be presumed to be dispersed in the amorphous state. At the higher ratio of 1:1 of drug to NS2 carriers, there was a small amount of crystalline IBU observed on the outer surface of the carriers, indicated by insignificant IBU characteristic peaks in Figure 3b.

**Figure 4:**
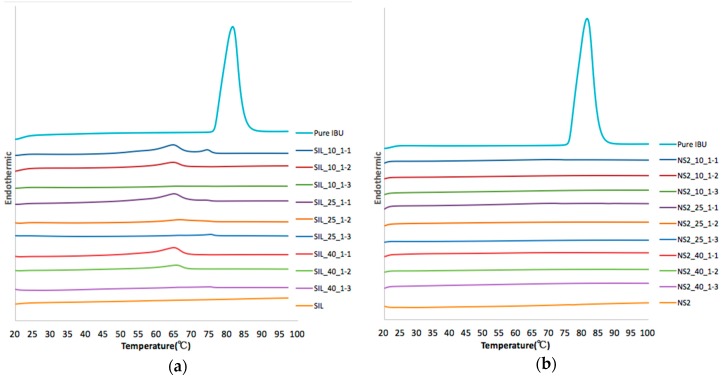
Differential scanning calorimetry (DSC) results of drug-loaded (**a**) S244FP and (**b**) NS2 under different drug loading conditions (refer to Table 1 for sample details).

In order to determine the percentage of the crystalline IBU in the drug-loaded samples, the DSC measurements were conducted for the IBU-loaded samples with the results shown in Figure 4. The crystalline IBU exhibited the melting transition peak at 77.5 °C. The measurements of the drug-loaded samples showed different endothermic transition behaviours with different carriers of S244FP and NS2 particles under the different loading conditions. In a previous study, it was shown that DSC measurements can also be used to distinguish the locations of the crystalline state of the loaded drug inside or outside the pores, in which the melting of crystalline IBU inside the pores was observed at lower temperatures compared to the melting of IBU outside the pores [17]. This was due to the smaller crystal size restricted by the pore structure inside the pores. In this study, at a higher loading ratio of 1:1 of drug to S244FP particles, two endothermic peaks observed at 64.7 and 74.9 °C were found. The second peak at 74.9 °C is suggested to be caused by the melting of the crystalline IBU on the outer surface of the carriers, while the first peak at 64.7 °C corresponds to the melting of the crystalline IBU in the inner pores of the carriers, indicating that although IBU was absorbed into the inner pores of the S244FP carriers, some of these drug molecules still remained in the crystalline form. Increasing the initial loading concentration from 10 to 25 mg/mL at the same drug to carrier ratio of 1:1, it was found that the second peak at 74.9 °C decreased in size in comparison with the first peak, indicating that more drug in the crystalline state had been loaded into the inner pores of the carriers relative to the surface. Upon decreasing the ratio of the drug to S244FP particles to 1:2, irrespective of the loading concentration, it was found that the first endothermic peak at 64.7 °C was both significantly bigger than the second peak, indicating that more crystalline IBU was present inside the pores of the carrier particles than at the surface, as well as significantly lower than that observed at a 1:1 ratio, indicating a relative reduction in crystalline IBU in the particle cores. At the lowest ratio of 1:3, the first peak disappeared, indicating that IBU was in the amorphous state in the inner pores of the carriers, and only a small fraction of crystalline IBU was observed on the outer surface of the carriers. Based on the calibration model developed in Section 4.2.4., the percentages of crystalline IBU were determined in Table 1, indicating that the overall percentages of crystalline IBU in the drug-loaded carriers decreased significantly with decreasing the drug to carrier ratio and was also less affected by the initial drug loading concentration.

The lack of crystalline IBU in the NS2 carriers at different loading conditions was also evident from the DSC thermographs shown in Figure 4b. There was only a very small endothermic peak detected for the drug-loaded samples at the drug and carrier ratio of 1:1, and the percentages of the crystalline IBU are also shown in Table 1. No peaks corresponding to any endo- or exo-thermic events were detected in the samples at the other loading conditions, indicating that all IBU molecules are in the amorphous state either on the outside or inside of the pores of the NS2 particles.

### 2.4. Dissolution Efficiency 

**Figure 5:**
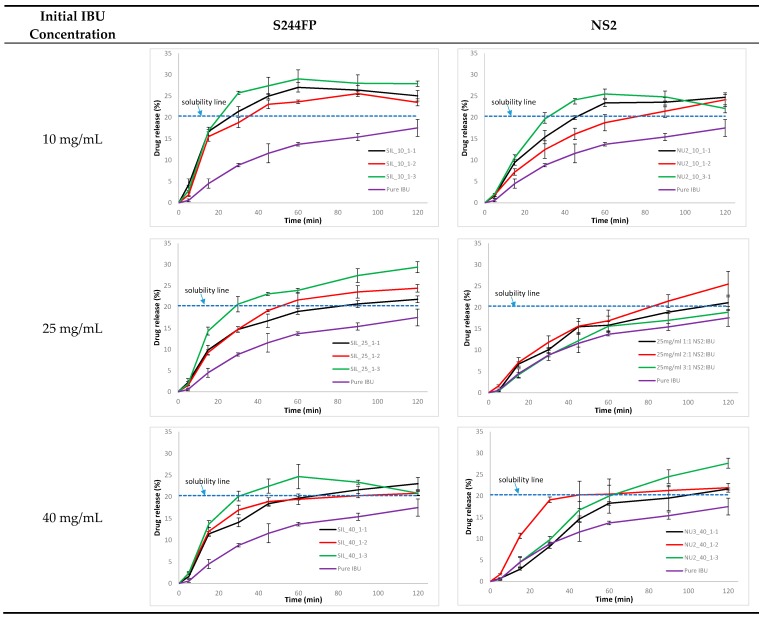
IBU dissolution profile from the drug-loaded carriers (refer to Table 1 for sample details).

The comparison of the IBU dissolution profiles from the drug-loaded carriers is shown in Figure 5. For the pure IBU, during a 120-minute dissolution, the concentration of IBU in the dissolution medium slowly reached 25 μg/mL, which was close to its theoretical solubility of 29 μg/mL [27]. A rapid initial drug release from the IBU-loaded S244FP samples was observed. At the same initial drug loading concentration, the samples generated by the lowest drug to carrier ratio of 1:3 resulted in the highest dissolution efficiency (D.E.) values shown in Table 1. Interestingly, when comparing the dissolution profiles of the samples generated at the same drug to carrier ratio, it was observed that a faster drug release profile was obtained for those at a lower initial drug loading concentration. It was found that dissolution from each of the IBU-loaded S244FP samples at the highest drug to carrier ratio of 1:1 after 30 min can lead to an IBU concentration in the dissolution medium that was at or above its solubility threshold, as shown in Figure 5. There is potential evidence that the supersaturation state was affected by the initial drug loading concentration in which the IBU-loaded samples at the lowest loading concentration of 10 mg/mL can generate the highest supersaturated drug concentrations. At the lowest drug to carrier ration of 1:3, only IBU-loaded S244FP samples at the loading concentration of 10 mg/mL appeared to achieve a supersaturated level during dissolution.

The improved IBU release rates from the NS2 carriers under different loading conditions were not significant in Figure 5. The loading factors of the initial drug concentration and the ratio of drug to carrier had little effect on the drug dissolution profiles. A moderate IBU supersaturated level in solution was only observed during the dissolution of the samples at the drug to carrier ratio of 1:1.

**Figure 6:**
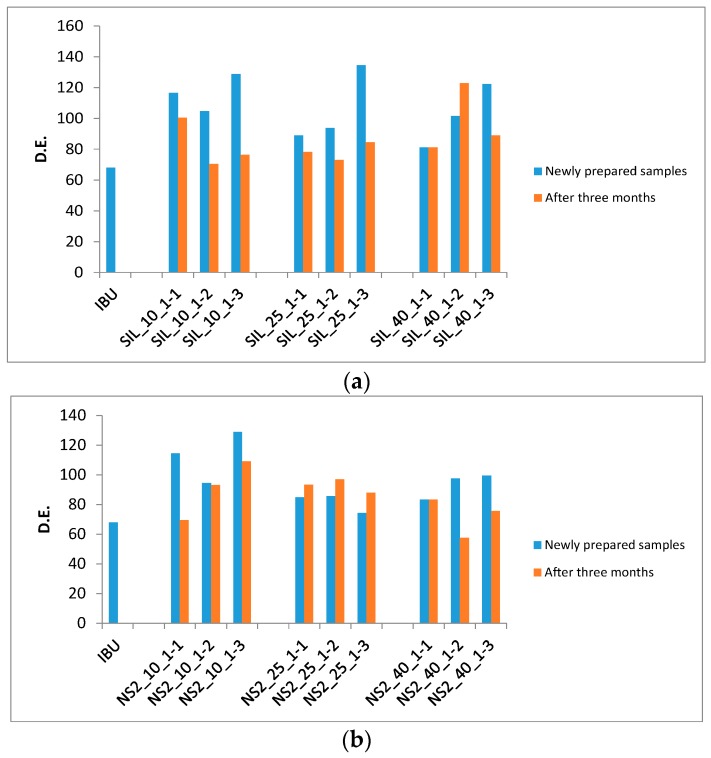
D.E. comparison of the drug-loaded samples (**a**) S244FP and (**b**) NS2.

The dissolution tests of the drug-loaded samples were repeated after three months of storage in sealed high density polyethylene (HDPE) bottles at room temperature. The comparison of the D.E. values of the drug-loaded samples is shown in Figure 6 and Table 1. It was found that dissolution rates from the S244FP carriers were reduced, and most of them were comparable with that of the pure IBU in Figure 6a. The change of the D.E. values of the drug-loaded NS2 samples was not significant in Figure 6b. The details of the dissolution profiles of the drug-loaded samples are shown in Figure S3 in the Supplementary Materials.

## 3. Discussion

It is known that mesoporous carriers have the ability to enhance the dissolution properties of poorly water-soluble drugs [2,3]. In this work, it has been shown that the enhanced drug dissolution depends on not only the properties of the porous carriers used, but also the loading processes. 

For the S244FP carriers, there was no evidence of chemical interaction between the host and loaded IBU shown in the FTIR results in Figure 2a; therefore, the loading factors played essential roles in the drug physical state and release kinetics. It was found that the ratio of the drug to carriers was the key factor to determine the physical state of the loaded drug in the carriers. A lower ratio of the drug to carriers can lead to more drugs in the amorphous form shown in Table 1. This can enhance the drug dissolution shown in Figure 5 in which at the same initial drug loading concentration, the highest D.E. of the drug-loaded samples was at the lowest ratio of 1:3. Different initial drug loading concentrations had little effect on the loaded drug physical state, resulting in a similar overall percentage of the crystalline IBU under the same the drug to carrier ratio. However, it did affect the location of the loaded IBU in the porous particles. This hypothesis was based on the fact that the melting of crystalline IBU inside the pores can be observed at lower temperatures with a broader peak compared to the melting of IBU with a higher temperature and a sharper peak outside the pores [17]. The study showed that a higher initial drug loading concentration can drive more crystalline drug in the inner pores of the carriers shown in the DSC results in Figure 4, leading to the reduced drug D.E. values in Figure 5 and Table 1. The drug release from the S244FP carriers showed a two-step drug release profile, which was in good agreement with previous reports [9]. It indicated that the IBU molecules located on the outer surface of the pores can be released quickly, whereas the drug molecules packed in the inner pores were released more slowly. In the meantime, the study showed that a supersaturation condition was generated from the drug-loaded S244FP carriers shown in Figure 5, which was affected by the initial drug loading concentration. A higher initial loading concentration can drive more drugs into the inner pores, leading to reduced dissolution rates and lower supersaturation, and in some cases, there was no supersaturation generated at all shown in Figure 5.

There was an interaction between the IBU and NS2 carriers shown in the FTIR results in Figure 2b. Therefore, the loading factors had little effect on the drug physical state and release kinetics. For example, even at a higher drug to carrier ratio, more than 95% of the drug was in the amorphous state. This could provide an effective way to create an amorphous form of IBU due to its low glass transition temperature of −42.3 °C [28]. Significantly lower desorption recovery rates from 40% to 54% were obtained from the IBU-loaded NS2 samples. This was consistent with the previous finding that incomplete desorption recovery was a common feature of an NS2-based delivery system for a diverse set of drugs [5]. Although IBU was in the amorphous form in the drug-loaded NS2 carriers, the IBU dissolution rate was not enhanced significantly in comparison with the pure IBU shown in Figure 5 and Table 1.

It is worth noting that in comparison with the NS2 carrier delivery systems, the dissolution rates of the drugs in the S244FP carriers can reduce after three months of storage, shown in Figure 6, indicating significant barriers would need to be addressed for the development of physically-stable formulations within the porous materials [10]. In the meantime, this study was based on a polar solvent of ethanol; however, that different solvents, in particular different nonpolar or polar solvents, could have profound effects on the drug loading efficiency and dissolution performance needs to be investigated in the future [29].

## 4. Materials and Methods

### 4.1. Materials

Ibuprofen (IBU, Fagron, Rotterdam, The Netherlands) was used as a model drug in this study. Silica Syloid^®^ 244FP (S244FP) and Neusilin^®^ US2 (NS2) were gifts from Grace GmbH & Co. (KG, Worms, Germany) and Fuji Chemical Industry Ltd. (Toyama, Japan). Methanol (HPLC grade) and ethanol (lab grade) were purchased from Fisher Scientific UK (Loughborough, UK). Double-distilled water was generated from a Bi-Distiller (WSC044.MH3.7, Fistreem International Limited, Loughborough, UK) and used throughout the study.

### 4.2. Methods

#### 4.2.1. Experimental Design and Drug Loading Procedure

A three-level design for each factor of the initial drug loading concentration (10, 25 and 40 mg/mL) and the ratio of the drug to carrier particles (1:1, 1:2 and 1:3) was used to study their effects on the physical state and dissolution profiles of IBU. The design of the experiments is shown in Table 1. The selection of the loading ratios (1:1, 1:2 and 1:3) of the drug to carriers was based on a preliminary study in which the drugs adsorbed either inside the pores or outside the surfaces of the carrier particles, and the amount of non-absorbed free drug in either crystalline or amorphous forms was insignificant. 

The synthesis process was summarized as: (1) dissolving pure IBU (500 mg) in an appropriate amount of ethanol in a round-bottom flask to prepare a concentrated solution of 10, 25 or 40 mg/mL; (2) adding carriers into the prepared IBU solution at a ratio of the drug and carriers of 1:1, 1:2 or 1:3 and then shaking evenly; (3) sealing the flask with a stopper for 24 h; (4) evaporating the solvent by a rotary evaporator (Büchi^®^ R-200, BUCHI UK Ltd., Chadderton, UK) for 15 min at 50 °C; (5) keeping the flask open in the fume cupboard overnight for complete elimination of the solvent.

#### 4.2.2. Polarized Light Microscopy 

The polarized optical microscopy images of the crystalline states of drug, carriers and drug-loaded samples were obtained by an optical microscope (Leica DM750, Leica Microsystems Limited, Heerbrugg, Switzerland) equipped with a lens of 10-time magnification, an infinity 2-1C camera and the Studio Capture Video microscopy software (Version 4.0, Mettler-Toledo Ltd., Leicester, UK).

#### 4.2.3. Fourier Transform Infrared Spectrometer 

FTIR tests were carried out using an ALPHA interferometer (Bruker UK, Limited, Coventry, UK) with a horizontal universal attenuated total reflectance (ATR) sample accessory. Samples were placed on the surface of the diamond ATR plate, and the ATR assembly was clamped to ensure good contact. In each of the measurements, 30 scans were collected per spectrum with a resolution of 2 cm^−1^ in the spectral region of 400–4000 cm^−1^ using OPUS software. All of the spectral data were collected at an ambient temperature between 20 and 23 °C.

#### 4.2.4. Differential Scanning Calorimetry and Its Calibration Model to Determine the Percentage of the Crystalline IBU in the Drug-Loaded Samples

DSC measurements were carried using a Perkin Elmer Jade DSC (PerkinElmer Ltd., Beaconsfield, UK). The temperature and heat flow of the instrument were calibrated using indium and zinc standards. A test sample (5–10 mg) was analysed in a crimped aluminium pan with a pinhole pierced lid. Measurements were carried out at a heating rate of 10 °C/min under a nitrogen flow rate of 20 mL/min.

**Figure 7:**
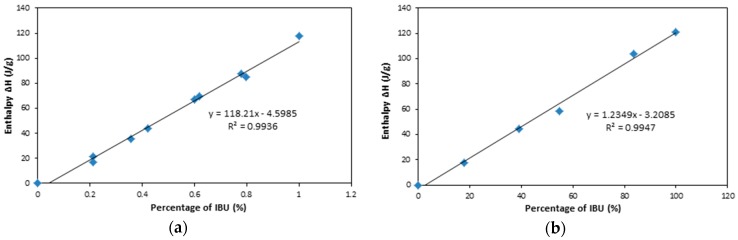
Relationship of the enthalpy and the percentage of IBU mixed with (**a**) S244FP and (**b**) NS2.

In order to determine the percentage of the crystalline IBU in the drug-loaded samples, the physical mixtures of pure IBU crystals with S224FP or NS2 powders at different percentages were analysed by DSC measurements. The relationship between the enthalpy ΔH of the mixture of IBU and S224FP/NS2 powders and the percentage of IBU crystals is shown in Figure 7, indicting a good line relationship. The validation of the predictive models is shown in Table 2, showing that the percentage of crystalline IBU can be accurately predicted.

#### 4.2.5. X-ray Powder Diffraction

The XRPD pattern of solids was recorded from 5° to 30° at a scanning rate of 0.3° (2θ) min^−1^ by a D2 PHASER diffractometer (Bruker UK Limited, Coventry, UK). Cu-Kβ radiation was used with a voltage of 30 kV and a current of 10 mA.

#### 4.2.6. Drug Desorption Recovery Rate Analysis

In order to determine the maximal percentage of the drug desorbed from the carriers, the drug-loaded samples of 10 mg were immersed in 10 mL of ethanol with vigorous stirring for 2 h at room temperature. The total amount of IBU recovered from the carriers was quantified by HPLC. The drug desorption recovery rate is calculated by [5]:

(1) $$\mathrm{DDRR}=\frac{M_{d}}{M_{R}}\times 100\%$$

where *M*_d_ is the mass of desorbed drug from the drug loaded samples and *M*_R_ is the mass of the theoretically-loaded drug. Each sample was tested in triplicate.

#### 4.2.7. Dissolution Test

Dissolution experiments were performed under non-sink conditions in pH 2.0 simulated gastric fluid buffers without pepsin [22]. The IBU-loaded samples (equivalent 50 mg of pure IBU based on the initial loading experiments) were weighted into a 600-mL beaker, and then 350 mL of media were added. If the amount of drug used in the release experiments dissolved completely, its concentration was around 5 times its solubility of 29 μg/mL of free ibuprofen [27]. Dissolution testing was carried out at 37 ± 0.5 °C with magnetic stirring at 125 rpm. The sampling intervals were set as 5, 15, 30, 45, 60, 90 and 120 min. Two millilitres of the sample were withdrawn at each sampling interval from the dissolution beaker and filtered by the Millipore filter (0.45-µm pore size) for analysis by HPLC. Each sample was tested in triplicate.

#### 4.2.8. High Performance Liquid Chromatography Analysis

The concentration of IBU in solution was analysed by the Perkin Elmer series 200 HPLC system. An HAISLL 100 C18 column (5 µm, 250 mm × 4.6 mm) (Higgins Analytical, Inc., Mountain View, CA, USA) at ambient temperature was used. The mobile phase was composed of 85% methanol and 15% water (with 0.5% formic acid), and the flow rate was 1.5 mL/min using an isocratic method. The detection UV wavelength was set as 220 nm. The injection volume was 20 µL.

#### 4.2.9. Dissolution Efficiency 

In order to evaluate the dissolution performance of the IBU-loaded samples at different conditions, the dissolution efficiency was calculated and compared as [30]:

(2) $$D.E.=\frac{\int_{t_{1}}^{t_{2}}y\cdot dt}{y_{100}\cdot \left(t_{2}-t_{1}\right)}\times 100\%$$

where y is the percentage of dissolved drug. D.E. is the area under the dissolution curve between time points $t_{1}$ and $t_{2}$ expressed as a percentage of the curve at maximum dissolution, $y_{100}$, over the same time point [31]. Normally, $t_{1}$ = 0 was set for powders, as the drugs release immediately. In this study, $t_{2}$ was set as 120 min, corresponding to the end of the dissolution tests. $y_{100}$ was set as a constant of 17.5%, which was the percentage of pure IBU powders dissolved in the dissolution test.

#### 4.2.10. Stability Test

In order to study the stability of the drug in the carriers, the drug-loaded samples were stored in the sealed HDPE bottles at room temperature for 3 months. Dissolution tests were repeated to evaluate the changes of the drug dissolution profiles.

## 5. Conclusions

In this study, drug-loaded mesoporous systems were prepared and characterised. The loading factors of the initial drug loading concentration and ratio of drug to carriers were studied for two different mesoporous carriers of S244FP and NS2. The study has shown that the dissolution rate of poorly water-soluble drug IBU can be improved significantly using the mesoporous S244FP carriers. The reason for the dissolution improvement was due to the transformation of crystalline IBU into the amorphous form when loaded into the mesoporous carriers. The quantity of the crystalline form of IBU in the carriers was influenced by the drug to carrier ratio, and the location of the crystalline IBU was affected by the initial drug loading concentration, both of which affected the drug dissolution profiles. Despite the proven dissolution advantages of the drug-loaded S244FP samples, there are still hurdles to be tackled before the application of the mesoporous materials as delivery systems, in particular the reduced dissolution efficiency following storage, presumably due to solid state conversion back to the crystalline form.

Due to the molecular interaction between the IBU and NS2 carriers, the loading factors had little effect on the drug physical state and release kinetics. The chemical interaction can also cause other problems, such as incomplete drug desorption and insignificant dissolution improvement from the NS2 carrier delivery systems. Therefore, care and extensive evaluation must be taken when NS2 is chosen as the carrier delivery systems.

## Figures and Tables

**Table 1:** Experimental design and characterization. IBU: Ibuprofen; S244FP: Syloid^®^ 244FP; NS2: Neusilin^®^ US2.

Carrier	Number	Loading Factors		IBU Crystalline Percentage (%)	Drug Desorption Recovery (%)	Dissolution Efficiency (D.E. %)		
		IBU Concentration (mg/mL)	Drug to Carrier Ratio			Newly Prepared Samples	Samples after 3-Month Storage	Difference (%)
S244FP	SIL_10_1-1	10	1:1	32.7	89.8 ± 3.3	116.5	100.3	16.1
	SIL_10_1-2	10	1:2	18.9	98.2 ± 1.4	104.6	70.4	34.2
	SIL_10_1-3	10	1:3	9.1	78.8 ± 2.6	128.8	76.2	52.5
	SIL_25_1-1	25	1:1	33.8	109.1 ± 1.1	88.9	78.1	10.7
	SIL_25_1-2	25	1:2	14.1	88.2 ± 4.7	93.8	72.9	21.0
	SIL_25_1-3	25	1:3	8.7	91.1 ± 1.2	134.6	84.4	50.2
	SIL_40_1-1	40	1:1	30.5	107.5 ± 3.6	111.6	111.6	0.0
	SIL_40_1-2	40	1:2	22.3	95.6 ± 2.2	101.4	122.9	−21.5
	SIL_40_1-3	40	1:3	6.8	100.1 ± 4.7	122.4	88.9	33.5
NS2	NS2_10_1-1	10	1:1	6.4	53.7 ± 2.7	114.6	69.5	45.1
	NS2_10_1-2	10	1:2	0	46.4 ± 0.3	94.4	93.1	1.3
	NS2_10_1-3	10	1:3	0	40.8 ± 1.3	128.9	109.2	19.7
	NS2_25_1-1	25	1:1	4.1	52.2 ± 0.7	84.8	93.3	−8.5
	NS2_25_1-2	25	1:2	0	45.2 ± 0.7	85.6	97.0	−11.4
	NS2_25_1-3	25	1:3	0	48.4 ± 1.6	74.4	87.9	−13.5
	NS2_40_1-1	40	1:1	0	54.1 ± 2.2	83.3	83.3	0.0
	NS2_40_1-2	40	1:2	0	53.5 ± 0.8	97.5	57.6	39.9
	NS2_40_1-3	40	1:3	0	46.9 ± 0.5	99.5	75.7	23.8

**Table 2:** Comparison of the predicted and actual concentrations.

Sample	Actual IBU Percentage (%)	Enthalpy ΔH (J/g)	Predicted IBU Percentage (%)	Prediction Error (%)
IBU and S244FP	75.23	83.13	74.21	1.35
	35.89	39.36	37.19	3.60
IBU and NS2	9.00	8.35	9.36	3.98
	24.86	28.14	25.39	2.11

## Supplementary Materials

The following are available online at www.mdpi.com/1996-1944/10/2/150/s1. Figure S1: Structures of (a) ibuprofen; (b) Silica SYLOID^®^ 244 FP and (c) Neusilin^®^ US2; Figure S2: Images of the ibuprofen (IBU)-loaded samples; Figure S3: Comparison of the dissolution profiles of the drug loaded samples of fresh and after three months of storage.
