# Supplementary material for: Investigating the Effects of Loading Factors on the In Vitro Pharmaceutical Performance of Mesoporous Materials as Drug Carriers for Ibuprofen
Source: Materials (Basel). 2017 Feb 9;10(2):150. doi: 10.3390/ma10020150 (PMC5459193; doi:10.3390/ma10020150)
Supplement: Supplementary file 1 [file materials-10-00150-s001.pdf]

Supplementary Materials: Investigating the Effects of Loading Factors on the In Vitro Pharmaceutical Performance of Mesoporous Materials as Drug Carriers for Ibuprofen

Junmin Lai, Wu Lin, Peter Scholes and Mingzhong Li

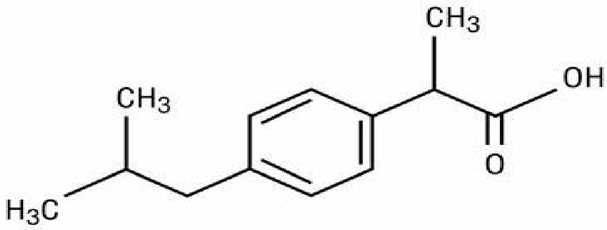

(**a**)

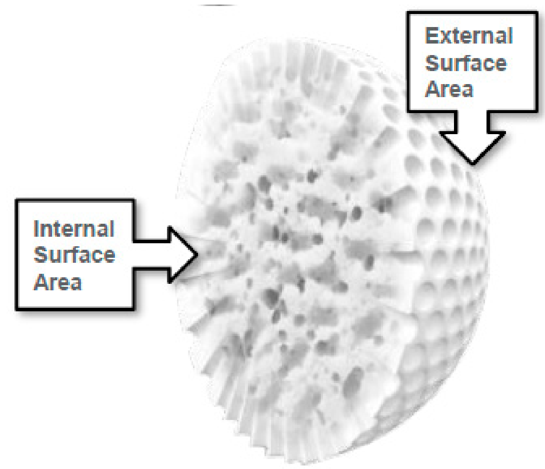

(**b**)

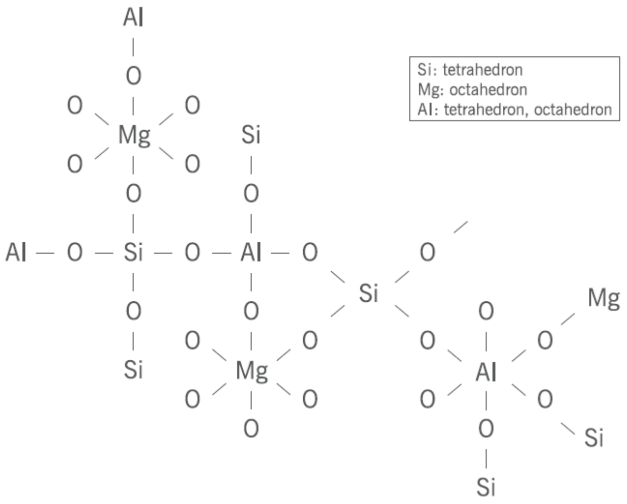

(**c**)

Figure S1. Structures of: (**a**) ibuprofen; (**b**) Silica SYLOID® 244FP; and (**c**) Neusilin® US2.

Initial IBU Concentration	Silica SYLOID® 244 FP	Neusilin® US2
10 mg/mL	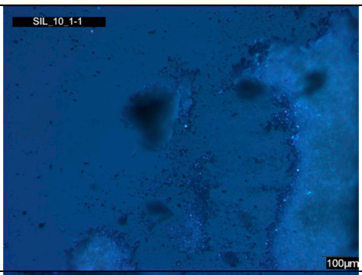	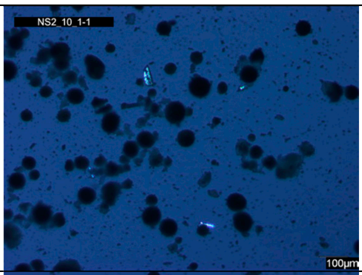
	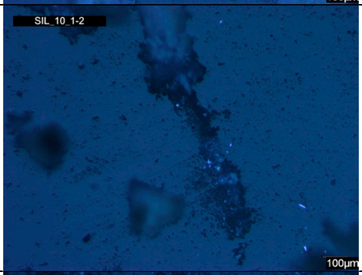	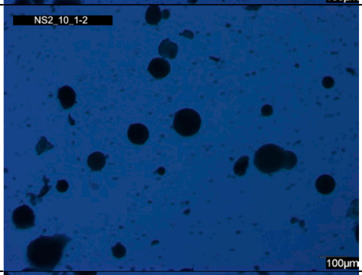
	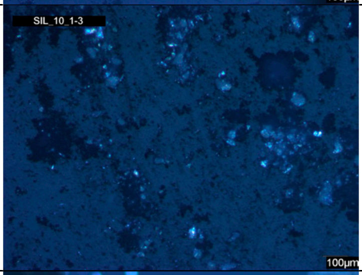	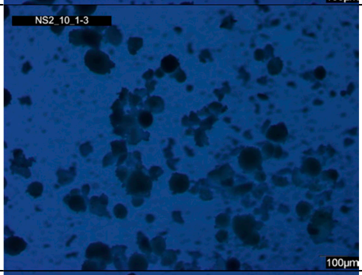
25 mg/mL	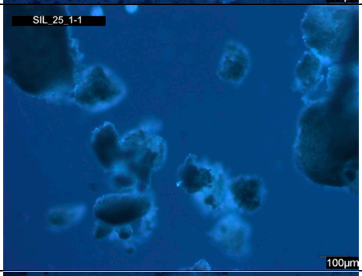	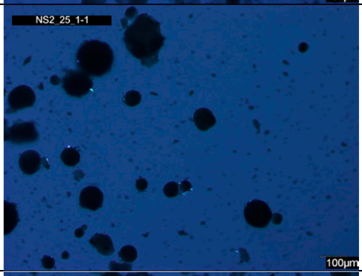
	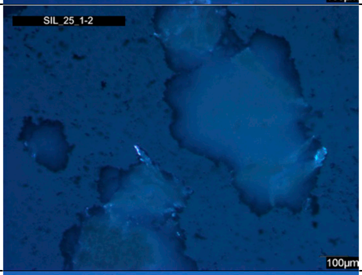	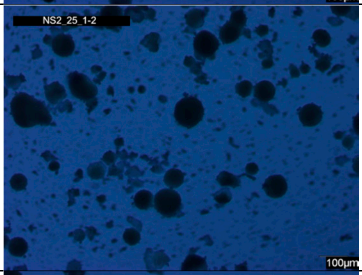
	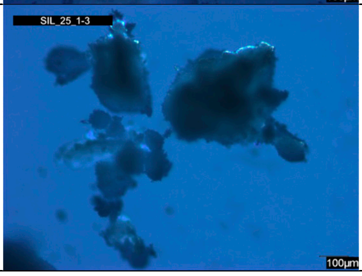	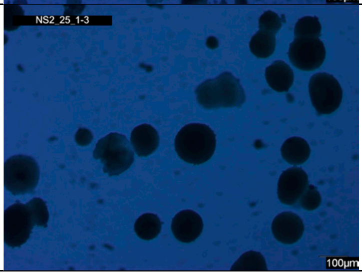

Figure S2. *Cont.*

40 mg/mL	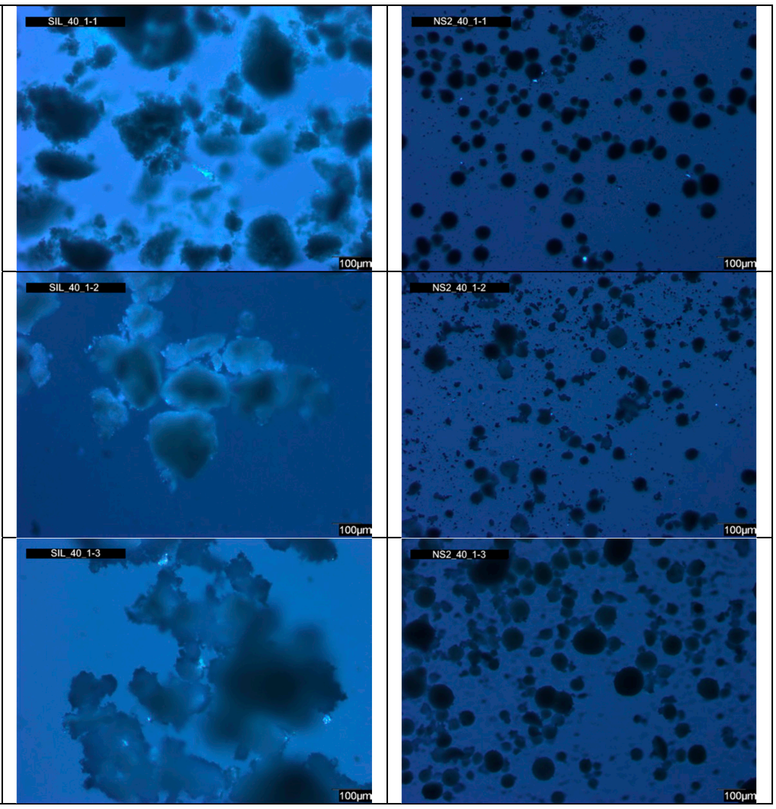

Figure S2. Images of the ibuprofen (IBU)-loaded samples.

Initial IBU Concentration	Silica SYLOID® 244 FP	Neusilin® US2
10 mg/mL	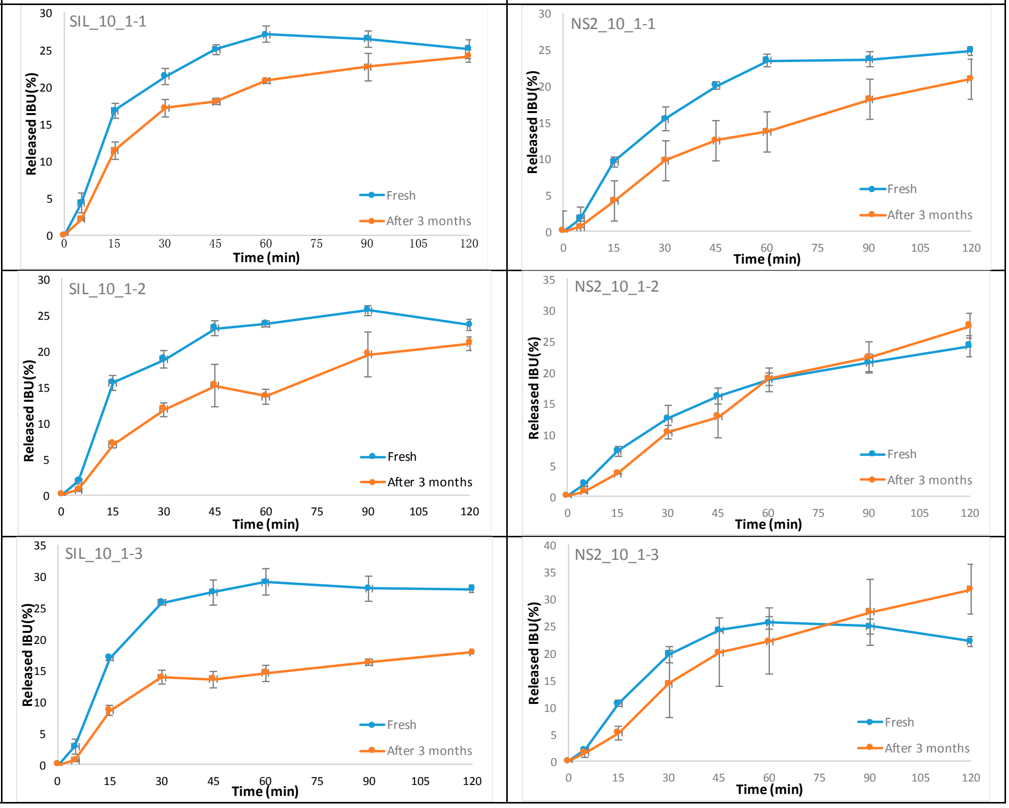	

Figure S3. *Cont.*

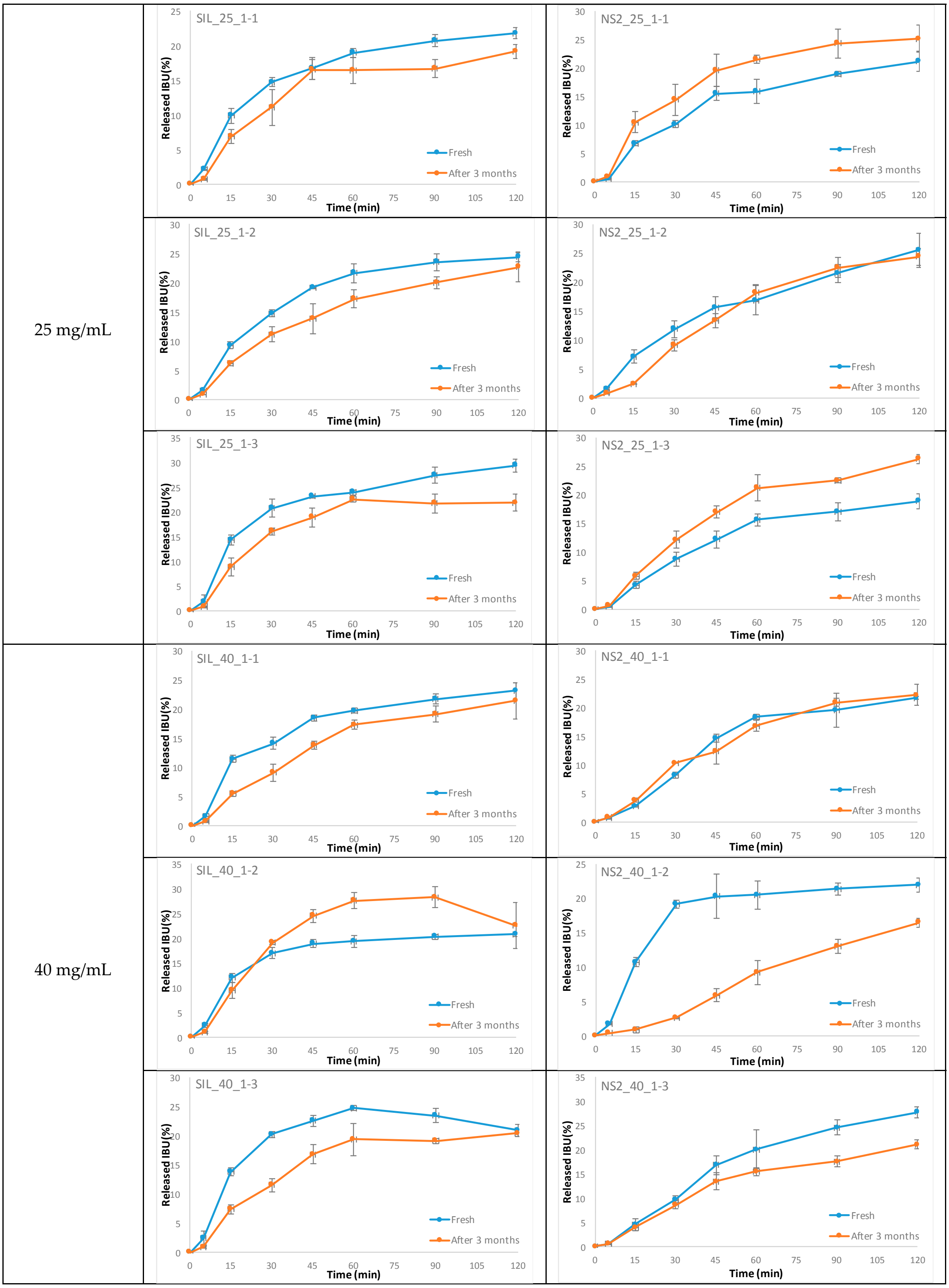

Figure S3. Comparison of the dissolution profiles of the drug-loaded samples of fresh and after three months of storage.
